# Modulating Ni/Ce Ratio in Ni*_y_*Ce_100−_*_y_*O*_x_* Electrocatalysts for Enhanced Water Oxidation

**DOI:** 10.3390/nano11020437

**Published:** 2021-02-09

**Authors:** Jun Yu, Qi Cao, Chen Qiu, Lei Chen, Jean-Jacques Delaunay

**Affiliations:** 1 Key Laboratory of Energy Thermal Conversion and Control, and Key Laboratory of Environmental Medicine Engineering of Ministry of Education, School of Energy and Environment, Southeast University, Nanjing 210096, China; yujun12@mails.tsinghua.edu.cn; 2 Guangdong Key Lab of Nano-Micro Materials Research, School of Chemical Biology and Biotechnology, Peking University Shenzhen Graduate School, Shenzhen 518055, China; qiuc12@163.com; 3 Graduate School of Engineering, The University of Tokyo, 7-3-1 Hongo, Bunkyo-ku, Tokyo 113-8656, Japan; 4 State Key Laboratory of Tribology, Tsinghua University, Beijing 100084, China

**Keywords:** oxygen evolution reaction (OER), Ni*_y_*Ce_100−*y*_O*_x_*, oxygen vacancies

## Abstract

Oxygen evolution reaction (OER) is the key reaction for water splitting, which is used for hydrogen production. Oxygen vacancy engineering is an effective method to tune the OER performance, but the direct relationship between the concentration of oxygen vacancy and OER activity is not well understood. Herein, a series of Ni*_y_*Ce_100−*y*_O*_x_* with different concentration of oxygen vacancies were successfully synthesized. The larger concentration of oxygen vacancies in Ni_75_Ce_25_O*_x_* and Ni_50_Ce_50_O*_x_* result in their lower Tafel slopes, small mass-transfer resistance, and larger electrochemical surface areas of the catalysts, which account for the higher OER activities for these two catalysts. Moreover, with a fixed current density of 10 mA/cm^2^, the potential remains stable at 1.57 V for more than 100 h, indicating the long-term stability of the Ni_75_Ce_25_O*_x_* catalyst.

## 1. Introduction

Hydrogen is considered as one of the clean energy to replace the traditional fossil fuel to solve the environmental and energy problem. Electrocatalytic water splitting is an effective way to produce hydrogen with high purity from intermittent renewable energy (i.e., wind and solar energy). Two half reactions including hydrogen evolution reaction (HER) and oxygen evolution reaction (OER) are at cathode and anode, respectively. The four-electron transfer steps of OER lead to the large sluggish kinetics, resulting in a high overpotential for water splitting [1,2,3,4,5,6,7]. An OER catalyst with high activity is of critical importance to reduce the overpotential so that a small bias voltage can be used and thus increase the efficiency of the OER process.

The OER activity of a catalyst can be enhanced by introducing defects, especially oxygen vacancies [8,9,10,11]. The local electron density distribution will be changed and the oxygen vacancy itself can be the OER active sites. Wang et al. [12] treated Co_3_O_4_ nanosheets by Ar-plasma to get rich oxygen vacancies. The specific activity of the Co_3_O_4_ nanosheets with oxygen vacancies was 10 times higher than that of the pristine Co_3_O_4_. Ceria is a nonstoichiometric material and the oxygen vacancies are easily formed due to the shift between Ce^3+^ and Ce^4+^ [13,14,15,16,17]. The oxygen vacancies in Ce-containing catalysts such as CeO_2_/Co_3_O_4_ accelerated the electron transfer, resulting in good OER activity [9]. In our previous work, oxygen vacancies in NiCeO*_x_* catalyst were proved to be the active sites for water oxidation [10]. However, the direct correlation between the concentration of oxygen vacancies and OER activity has not been identified yet.

In this work, a series of Ni*_y_*Ce_100−*y*_O*_x_* with different Ni/Ce ratio were synthesized on NF (nickel foam)/NiO substrate with simple dip-coating and annealing methods. The surface NiO obtained from the oxidation of NF can prohibit the diffusion of Ni atoms to the deposited Ni*_y_*Ce_100−*y*_O*_x_* so that the Ni/Ce ratio is not modified. Oxygen vacancy defects are formed successfully in all the NF/NiO/Ni*_y_*Ce_100−*y*_O*_x_* (simply referred to as Ni*_y_*Ce_100−*y*_O*_x_*) catalysts. The concentration of oxygen vacancy defects for Ni_75_Ce_25_O*_x_* and Ni_50_Ce_50_O*_x_* catalysts are larger than other Ni*_y_*Ce_100−*y*_O*_x_* catalysts, resulting in a similar larger electrochemically active surface area and the same lower Tafel slope of 66 mV/decade. The overpotential to achieve a current density of 10 mA/cm^2^ for the Ni_75_Ce_25_O*_x_* and Ni_50_Ce_50_O*_x_* catalysts are 338 mV and 341 mV, respectively. It is noted that these overpotentials are lower than other Ni*_y_*Ce_100−*y*_O*_x_* catalysts. With a fixed current density of 10 mA/cm^2^, the Ni_75_Ce_25_O*_x_* catalyst exhibits an ultra-high stability of over 100 h.

## 2. Materials and Methods

### 2.1. Sample Synthesis

The synthesis of NF/NiO/Ni*_y_*Ce_100−*y*_O*_x_* catalysts. The first step was to prepare the NF/NiO substrate. A 10 × 15 mm^2^ Nickel Foam (NF, >99.99%, MTI Corporation, Richmond, CA, USA) substrate with a thickness of 0.08 mm was firstly cleaned by acetone (99%, Wako) in an ultrasonic bath for 5 min. Then the NF was rinsed by deionized water three times. Subsequently, the NF was dried in air and annealed for 2 h with a heating rate of 2 °C/min at 400 °C in a muffle furnace to obtain NF/NiO substrate. The second step is to prepare the nickel and cerium mixed precursor solution. The precursor solution was prepared by dissolving 0.3 M citric acid and 0.15 M metal ions in 20 mL ethanol. The molar ratio of Ni and Ce ions were 95:5, 90:10, 75:25, 50:50, 25:75, and 10:90 for the catalysts of Ni_95_Ce_5_O*_x_*, Ni_90_Ce_10_O*_x_*, Ni_75_Ce_25_O*_x_*, Ni_50_Ce_50_O*_x_*, Ni_25_Ce_75_O*_x_* and Ni_10_Ce_90_O*_x_*, respectively. Ce(NO_3_)_3_ 6H_2_O offers the Ce ions and Ni(NO_3_)_2_ 6H_2_O offers the Ni ions. The final step is the preparation of NF/NiO/Ni*_y_*Ce_100−*y*_O*_x_* catalysts, hereafter simply referred to as the Ni*_y_*Ce_100−*y*_O*_x_*. The prepared precursor solution was deposited onto NF/NiO by dip coating and then annealed in the same manner as the NF/NiO substrate.

### 2.2. Structural Characterization

A field emission Scanning electron microscope (SEM, JEOL JSM 7600 FA) was used for the measurements of SEM. A diffractometer (Rigaku Co. Ltd., SmartLab, Japan) with Cu Kα radiation (dwelling time = 2 s, incident angle = 0.5°, step size = 0.02°, λ = 1.541 Å) was used for the collecting of grazing incidence X-ray diffraction data. A Renishaw inVia Raman Microscope system was used to acquire the Raman spectra at room temperature (25 °C). A ×100 objective and a 532 nm excitation laser were used. A PHI 5000 VersaProbe (ULVAC-PHI) with an Al K*α* X-ray source (1486.6 eV) was used to obtain the X-ray photoelectron spectroscopy (XPS). The pass energies of 117.4 eV and 23.5 eV were used for the electron analyzer to analyze the wide scans and narrow scans, respectively.

### 2.3. Electrochemical Measurements

A cylindrical glass cell with a standard three-electrode configuration was used for the electrochemical measurements. A Pt wire and a Ag/AgCl electrode were used as the counter electrode and the reference electrode, respectively. The working electrode was Ni*_y_*Ce_100−*y*_O*_x_* electrode. A potentiostat (Princeton Applied Research, VersaSTAT 4) was used to perform the electrochemical measurements. The potentials were calibrated against the RHE according to the following equation: (*E*_RHE_ = *E*_Ag/AgCl_ + 0.059 pH + *E*^0^_Ag/AgCl_), where *E*_Ag/AgCl_ is the potential difference measured between the Ag/AgCl electrode and the working electrode, *E*^0^_Ag/AgCl_ (0.1976 V at 25 °C) is the standard electrode potential for an Ag/AgCl electrode, pH is the pH of the electrolyte solution, and *E*_RHE_ is the calibrated potential. The electrolytes used were saturated with oxygen before and during the OER experiments.

The polarization curves were collected through linear sweep voltammetry (LSV), and the scan rate was 10 mV/s. Controlled-current water electrolysis was performed using a chronopotentiometric technique [16]. The solution resistance R*_s_* (~2 Ω), determined using the electrochemical impedance spectroscopy (EIS) technique [16], was used to correct the *iR* drop across the solution. Unless otherwise stated, all given potentials are vs. RHE and corrected for the *iR* drop across the electrolyte. Tafel plots obtained from the steady-state polarization curves with a scan rate of 1 mV/s. Cyclic voltammetry (CV) was used to determine the electrochemical capacitance of the samples presented in this paper [18]. The potential was swept in a range from 0.05 V above the open-circuit potential (OCP) to 0.05V below the OCP in a static solution with five different scan rates: 0.005, 0.01, 0.025, 0.05 and 0.1 V s^−1^. The working electrode was held for 10 s at each end of the potential sweep before continuing to the next sweep. All experiments were performed at room temperature.

## 3. Results and Discussion

**Figure 1:**
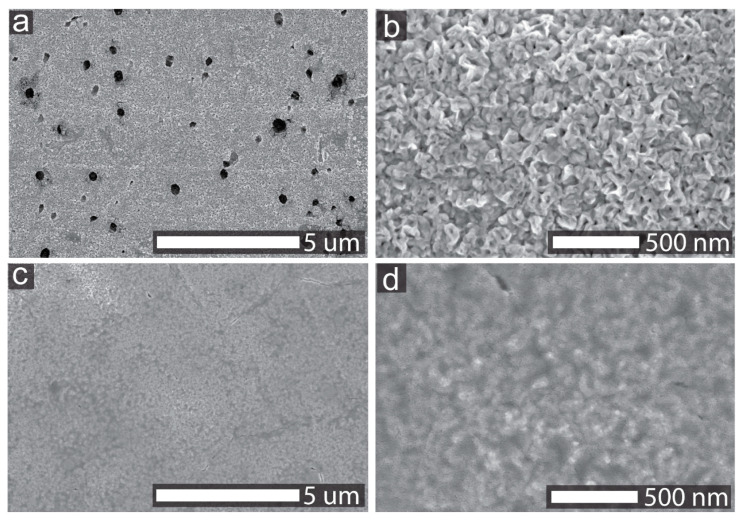
SEM images of NF/NiO (**a**,**b**) and Ni_95_Ce_5_O*_x_* (**c**,**d**) samples.

The surface morphology information of the NF/NiO and Ni*_y_*Ce_100−*y*_O*_x_* samples was analyzed by SEM, and the images are shown in Figure 1. Holes with sizes varying from several nanometers to a few hundred nanometers were observed on the surface of NF/NiO. NF/NiO sample was prepared by annealing Ni substrate in the muffle furnace for 2 h with the elevating rate of 2 °C/min, and these holes were from the Ni foam substrate. The surface of NF/NiO consisted of small nanocrystals, as shown in Figure 1b. The Ni*_y_*Ce_100−*y*_O*_x_* samples were synthesized by depositing nickel and cerium mixed precursor solutions with different Ni/Ce ratios on NF/NiO and then annealing the samples in air. The deposited layer covers the surface of NF/NiO nanocrystals for the Ni_95_Ce_5_O*_x_* sample, as shown in Figure 1c,d. Other Ni*_y_*Ce_100−*y*_O*_x_* catalysts have the similar morphologies with the Ni_95_Ce_5_O*_x_* catalyst, as displayed in Figure S1 of the supporting information.

**Figure 2:**
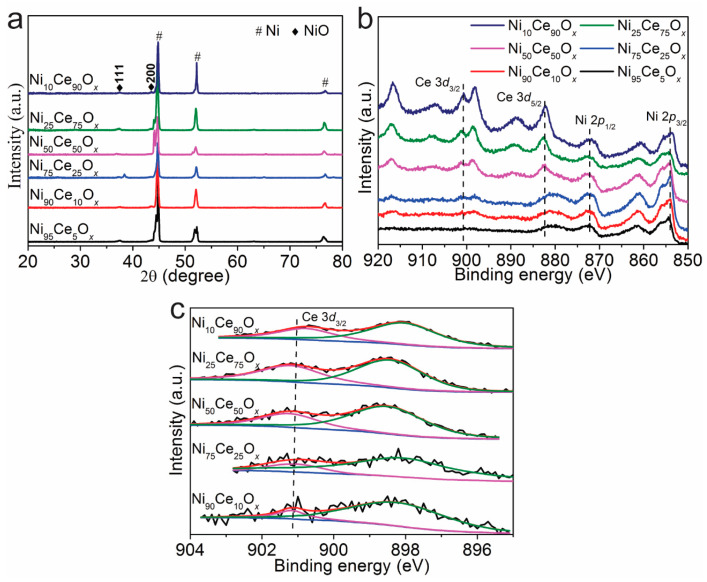
(**a**) X-ray diffraction patterns and (**b**,**c**) XPS spectra of Ni*_y_*Ce_100−*y*_O*_x_* samples.

Grazing incidence XRD measurements were performed to examine the crystal structure of the Ni*_y_*Ce_100−*y*_O*_x_* samples, and the results are shown in Figure 2a. Only Ni and NiO related peaks were found and no CeO_2_ related peaks were detected for Ni*_y_*Ce_100−*y*_O*_x_* samples. It indicates that Ni and Ce mixed uniformly in the top layer and formed an amorphous structure. The surface element information of the Ni*_y_*Ce_100−*y*_O*_x_* samples was further analyzed by XPS. As shown in Figure 2b, Ce 3d peaks of CeO_2_ and Ni 2p peaks of NiO were observed and indicates that the deposited layers of Ni*_y_*Ce_100−*y*_O*_x_* catalysts are indeed composed of Ni and Ce mixed oxides. The curve fitting results of Ce 3d_3/2_ for Ni*_y_*Ce_100−*y*_O*_x_* samples are shown in Figure 2c. The peak areas of Ce 3d_3/2_ for Ni_90_Ce_10_O*_x_*, Ni_75_Ce_25_O*_x_*, Ni_50_Ce_50_O*_x_*, Ni_25_Ce_75_O*_x_* and Ni_10_Ce_90_O*_x_* samples are 31.7, 55.7, 206.2, 433.0 and 603.3. For Ni*_y_*Ce_100−*y*_O*_x_* samples, the peak areas of Ce 3d_3/2_ are in proportion with the Ce content, and the higher Ce content will lead to larger peak areas of Ce 3d_3/2_. The sequence of the peak areas of Ce 3d_3/2_ of Ni*_y_*Ce_100−*y*_O*_x_* samples are in accordance with the Ce content in these samples (Table S1). This indicates that the designed Ni/Ce ratios remain constant for the synthesized Ni*_y_*Ce_100−*y*_O*_x_* samples.

**Figure 3:**
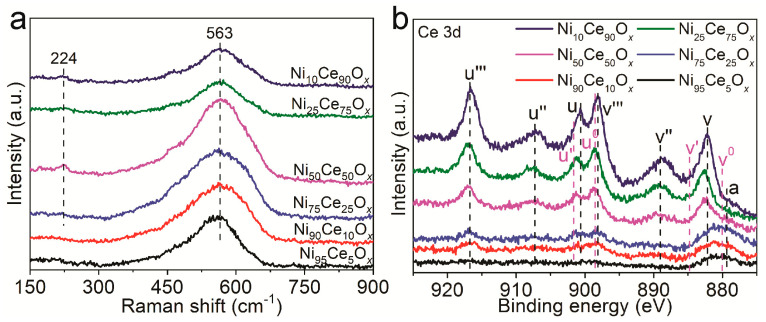
(**a**) Raman spectra and (**b**) XPS spectra of Ni*_y_*Ce_100−*y*_O*_x_* samples. The peak labeled *a* in the XPS spectra is ascribed to the Ni 2p peak.

Two Raman peaks of 224 cm^−1^ and 563 cm^−1^ were observed for Ni_75_Ce_25_O*_x_*, Ni_50_Ce_50_O*_x_*, Ni_25_Ce_75_O*_x_* and Ni_10_Ce_90_O*_x_* samples, as shown in Figure 3a. For Ni_95_Ce_5_O*_x_* and Ni_90_Ce_10_O*_x_* samples, only the peak of 563 cm^−1^ was observed. Crystalline CeO_2_ is known to have a strong F_2g_ Raman peak at 464 cm^−1^ related to its fluorite structure [19]. The presence of ions with the oxidation states lower than Ce^4+^ in the CeO_2_ has been shown to induce a Raman band, known as the D band, from 500 to 700 cm^−1^ [19,20,21]. This band is associated with the presence of oxygen vacancy defects created in the non-stoichiometric CeO_2-*y*_ by the 3+ coordinated ions. In addition to the introduction of the D band, the F_2g_ band will be weakened and becomes asymmetric and broad [22]. In the Raman spectra of the Ni*_y_*Ce_100−*y*_O*_x_* samples, there is no F_2g_ band, which suggests that there is no crystalline CeO_2_ with a fluorite structure in these samples [23]. The broad peak at 563 cm^−1^ (D band) indicates the formation of oxygen vacancy defects. The oxygen vacancy defects should be related to the presence of Ce^3+^ because of the incorporation of Ni into CeO_2_, as suggested by the literature [19,20,23]. Furthermore, the amorphous structure of Ni*_y_*Ce_100−*y*_O*_x_* contributes to the broadness of the peak [22,23]. According to the areas of this peak among different Ni*_y_*Ce_100−*y*_O*_x_* samples, we can roughly estimate the concentration of oxygen vacancy defects in these catalysts. The peak areas of the D band have been calculated and summarized in Table S2. The peak areas of 563 cm^−1^ for Ni_75_Ce_25_O*_x_* and Ni_50_Ce_50_O*_x_* samples are similar and larger than those of other Ni*_y_*Ce_100−*y*_O*_x_* samples, suggesting that these two catalysts own larger concentration of oxygen vacancy defects. The peak at 224 cm^−1^ is related to Ce-OH vibrations which are resulted from surface defects. Different types of hydroxyl groups generated by the dissociation of surface adsorbed water and doubly bridging hydroxyl groups on reduced cerium oxide are detected in the Raman spectra [24].

XPS was carried out on Ni*_y_*Ce_100−*y*_O*_x_* samples to further analyze the oxygen vacancy defects. The Ce 3d peaks of CeO_2_ were observed for Ni*_y_*Ce_100−*y*_O*_x_* samples, as shown in Figure 3b. The Ce 3d band is composed of ten individual peaks, that are labeled on Figure 3b as *v*, *v″*, *v‴*, *u*, *u″*, *u‴*, *v^0^*, *v’*, *u^0^* and *u’*. The *v*, *v″*, *v‴*, *u*, *u″* and *u‴* peaks represent the 3d^10^4f^0^ state of Ce^4+^, and the *v^0^*, *v’*, *u^0^* and *u’* peaks represent the 3d^10^4f^1^ state of Ce^3+^ [13,25]. The intensities of these peaks increased with the increasing Ce content in Ni*_y_*Ce_100−*y*_O*_x_* samples. The concentration of Ce^3+^ and Ce^4+^ can be estimated according to the relative areas of the corresponding peaks. The major valence state of Ce was 4+, Ce^3+^ were also detected for Ni*_y_*Ce_100−*y*_O*_x_* samples. It is commonly known that the oxygen vacancy defects will be formed with the appearance of Ce^3+^ to maintain electrostatic balance according to Equation (1):

4Ce^4+^ + O^2−^ → 4Ce^4+^ + 2e^−^/☐ + 0.5O_2_ → 2Ce^4+^ + 2Ce^3+^ + ☐ + 0.5O_2_ (1)

☐ represents the empty position by the removal of O^2–^ from the lattice (i.e., oxygen vacancy defect). It suggests that the oxygen vacancy defects formed for the Ni*_y_*Ce_100−*y*_O*_x_* samples, which is consistent with the Raman results.

**Figure 4:**
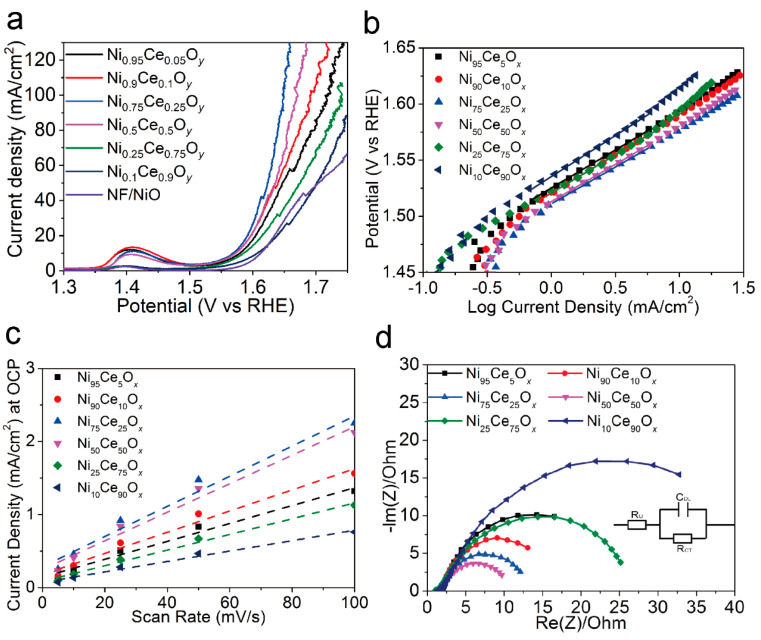
(**a**) Polarization curves of the NF/NiO substrate and Ni*_y_*Ce_100−*y*_O*_x_* catalysts for the OER with a scan rate of 10 mV/s. (**b**) Tafel plots obtained from the steady–state polarization curves with a scan rate of 1 mV/s. (**c**) Current density at OCP vs. CV scan rate for Ni*_y_*Ce_100−*y*_O*_x_* samples. The slope of current density at OCP vs. scan rate stands for the double–layer capacitance. (**d**) Nyquist plots of Ni*_y_*Ce_100−*y*_O*_x_* samples obtained at 1.55 V vs. RHE. The inset is the electrical equivalent circuit.

To test the electrochemical performance of Ni*_y_*Ce_100−*y*_O*_x_* catalysts, the polarization curves of these catalysts (Figure 4a) were obtained using Linear Sweep Voltammetry (LSV). The overpotentials of the catalysts for the current density of 10 mA/cm^2^ are listed in Table 1. The Ni_10_Ce_90_O*_x_* catalyst showed the lowest current density for the applied potentials and had the largest overpotential of 363 mV for the current density of 10 mA/cm^2^. The overpotentials for the Ni_75_Ce_25_O*_x_* and Ni_50_Ce_50_O*_x_* catalysts were 338 mV and 341 mV to obtain the current density of 10 mA/cm^2^, which were lower than that of other samples. The Tafel slope results of Ni*_y_*Ce_100−*y*_O*_x_* catalysts were shown in Figure 4b and Table 1. The Tafel slopes of the Ni_75_Ce_25_O*_x_* and Ni_50_Ce_50_O*_x_* catalysts (66 mV/decade) were close to that of Ni_95_Ce_5_O*_x_*, Ni_90_Ce_10_O*_x_* and Ni_25_Ce_75_O*_x_* catalysts (68 mV/decade), and were lower than that of the Ni_10_Ce_90_O*_x_* catalyst (73 mV/decade). An electrocatalyst with a low Tafel slope will have a small kinetic barrier for electron and mass transfer [2,5]. This indicates that the transfer barriers of electron and mass in the Ni_75_Ce_25_O*_x_* and Ni_50_Ce_50_O*_x_* catalysts are slightly improved.

**Figure 5:**
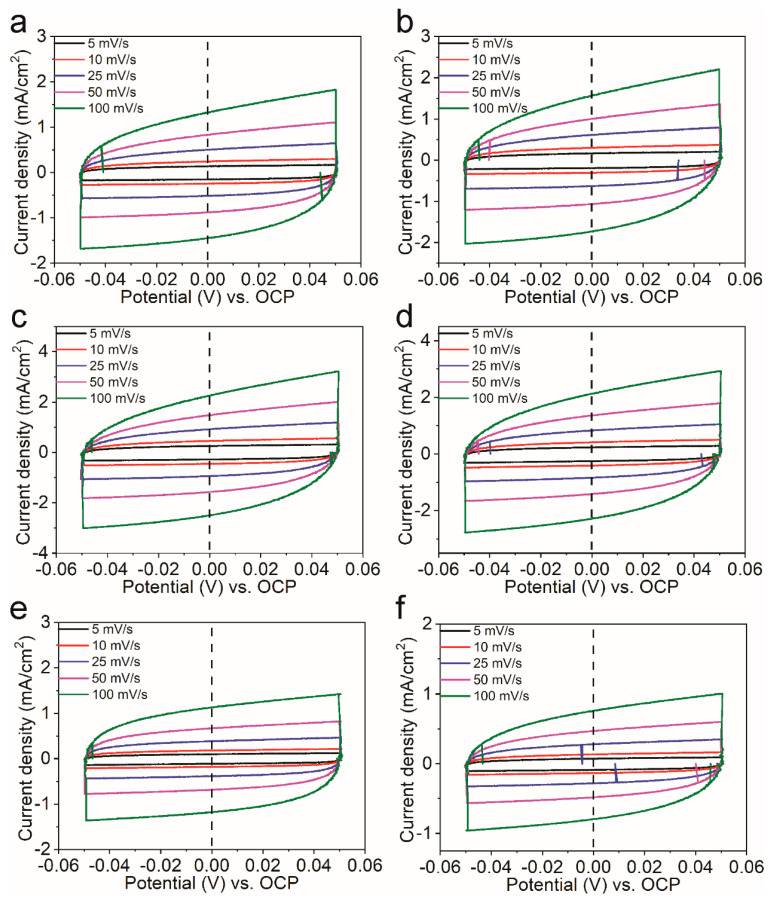
Cyclic voltammograms of (**a**) Ni_95_Ce_5_O*_x_*, (**b**) Ni_90_Ce_10_O*_x_*, (**c**) Ni_75_Ce_25_O*_x_*, (**d**) Ni_50_Ce_50_O*_x_*, (**e**) Ni_25_Ce_75_O*_x_* and (**f**) Ni_10_Ce_90_O*_x_* catalysts tested in a region with non–Faradaic process of the voltammogram with the scan rate of 5 mV/s, 10 mV/s, 25 mV/s, 50 mV/s and 100 mV/s. The value of the open circuit potential was 1.27 V vs. RHE.

The double-layer capacitance can be used to estimate the electrochemically active surface area (ECSA) of each sample. In order to know the double-layer capacitance, we first obtained CV curves of the capacitance current in the non-Faradaic voltage region (a 0.1 V potential range centered on the OCP) for several different scan rates (Figure 5). The rate of change in the current at OCP with respect to the scan rate corresponds to the double-layer capacitance [18]. For this reason, the current at OCP was plotted against the scan rate for the Ni*_y_*Ce_100−*y*_O*_x_* catalysts, and a line of best fit was fitted for each catalyst’s data set, as shown in Figure 4c. The double layer capacitance was 12.3 mF, 14.5 mF, 20.6 mF, 19.5 mF, 10.7 mF, and 7.1 mF for the Ni_95_Ce_5_O*_x_*, Ni_90_Ce_10_O*_x_*, Ni_75_Ce_25_O*_x_*, Ni_50_Ce_50_O*_x_*, Ni_25_Ce_75_O*_x_*, and Ni_10_Ce_90_O*_x_* samples, respectively. The ECSA can be calculated according to the formula ECSA = C_DL_/C_s_, where a specific capacitance of C_s_ = 0.040 mF cm^−2^ was used in this work [18]. The calculated ECSA values for the Ni*_y_*Ce_100−*y*_O*_x_* catalysts as well as other relevant electrochemistry parameters are summarized in Table 1. The ECSAs of the Ni_75_Ce_25_O*_x_* and Ni_50_Ce_50_O*_x_* catalysts are similar and larger than that of other Ni*_y_*Ce_100−*y*_O*_x_* catalysts. This is consistent with the observed current densities, as a larger ECSA means a sample has more active sites and therefore can catalyze more reactions at once and sustain a large current.

The charge transfer resistance (*R*_CT_) of the Ni*_y_*Ce_100−*y*_O*_x_* catalysts were obtained from their Nyquist plots, as shown in Figure 4d. As shown in Table 1, the *R*_CT_ of the Ni*_y_*Ce_100−*y*_O*_x_* catalysts decreased firstly and then increased with the increasing Ce content in the catalysts, and the Ni_50_Ce_50_O*_x_* catalyst had the lowest *R*_CT_ of 7.5 Ω at an applied bias of 1.55 V vs. RHE. The Ni_75_Ce_25_O*_x_* catalyst also showed a low *R*_CT_ of 9.9 Ω. However, the *R*_CT_ of the Ni_10_Ce_90_O*_x_* (31.8 Ω) catalyst was much higher than other Ni*_y_*Ce_100−*y*_O*_x_* catalysts. The small mass-transfer resistance of the Ni_75_Ce_25_O*_x_* and Ni_50_Ce_50_O*_x_* catalysts stands for their favorable OER kinetics.

For the Ce-based catalysts, the oxygen mobility can be promoted by the generated oxygen vacancy defects, resulting an improved ionic conductivity [10]. Also, the oxygen vacancy defects can be act as the OER active sites to catalyze the water oxidation reaction [17]. Therefore, the larger concentration of oxygen vacancy defects results in the lower Tafel slopes, small mass-transfer resistance, and larger ECSAs of the Ni_75_Ce_25_O*_x_* and Ni_50_Ce_50_O*_x_* catalysts, which account for the higher OER activities for these two catalysts.

**Figure 6:**
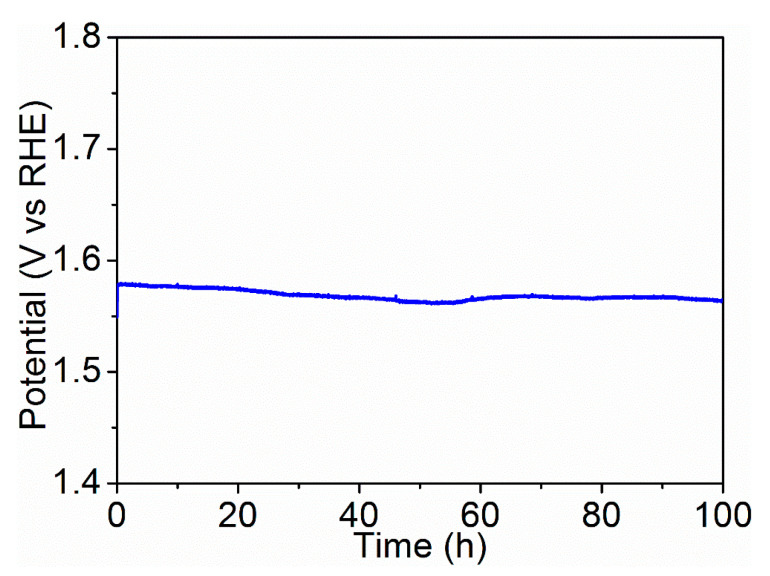
Potential trace of the Ni_75_Ce_25_O*_x_* sample obtained by fixing the current density for electrolysis at 10 mA/cm^2^. The electrolyte was 1 M KOH (pH ≈ 14).

Controlled-current water electrolysis (Figure 6) was done to test the long-term performance and stability of the Ni_75_Ce_25_O*_x_* catalyst. With a fixed current density of 10 mA/cm^2^, the potential remained stable at 1.57 V vs. RHE for more than 100 h. This indicates that the Ni_75_Ce_25_O*_x_* catalyst is very stable during long-term water electrolysis.

## 4. Conclusions

In summary, a series of NF/NiO/Ni*_y_*Ce_100−*y*_O*_x_* catalysts were synthesized through the simple dip-coating and annealing methods. The oxygen vacancy defects are formed successfully in all the Ni*_y_*Ce_100−*y*_O*_x_* catalysts, and the concentration of oxygen vacancy defects for Ni_75_Ce_25_O*_x_* and Ni_50_Ce_50_O*_x_* catalysts are larger than other Ni*_y_*Ce_100−*y*_O*_x_* catalysts. This results in the larger electrochemically active surface areas for Ni_75_Ce_25_O*_x_* and Ni_50_Ce_50_O*_x_* catalysts because of the abundant active sites offered by the defects. The rich oxygen vacancy defects also improve the ionic conductivity so that a lower Tafel slope of 66 mV/decade is obtained for Ni_75_Ce_25_O*_x_* and Ni_50_Ce_50_O*_x_* catalysts. The improved ionic conductivity also results in the small mass-transfer resistance of Ni_75_Ce_25_O*_x_* and Ni_50_Ce_50_O*_x_* catalysts, which is favorable for their OER kinetics. Therefore, the Ni_75_Ce_25_O*_x_* and Ni_50_Ce_50_O*_x_* catalyst exhibit higher OER activity than other Ni*_y_*Ce_100−*y*_O*_x_* catalysts with the overpotential of 338 mV and 341 mV for the current density of 10 mA/cm^2^. With a fixed current density of 10 mA/cm^2^, the potential remains stable at 1.57 V for more than 100 h, indicating the long-term stability of the Ni_75_Ce_25_O*_x_* catalyst.

## Figures and Tables

**Table 1:** Electrochemically active surface area (ECSA), Tafel slope, mass-transfer resistance (*R*_CT_) and the overpotential (*η*) for the current density of 10 mA/cm^2^ for each catalyst investigated in 1 M KOH.

Catalyst	ECSA/cm^2^	Tafel Slope	*R*_CT_	*η* for 10 mA/cm^2^
Ni_95_Ce_5_O*_x_*	307.5 cm^2^	68 mV/decade	18.2 Ω	351 mV
Ni_90_Ce_10_O*_x_*	362.5 cm^2^	68 mV/decade	12.6 Ω	350 mV
Ni_75_Ce_25_O*_x_*	515 cm^2^	66 mV/decade	9.9 Ω	338 mV
Ni_50_Ce_50_O*_x_*	487.5 cm^2^	66 mV/decade	7.5 Ω	341 mV
Ni_25_Ce_75_O*_x_*	267.5 cm^2^	68 mV/decade	21 Ω	356 mV
Ni_10_Ce_90_O*_x_*	177.5 cm^2^	73 mV/decade	31.8 Ω	363 mV

## Supplementary Materials

The following are available online at https://www.mdpi.com/2079-4991/11/2/437/s1, Figure S1: SEM images of Ni_90_Ce_10_O*_x_*, Ni_75_Ce_25_O*_x_*, Ni_50_Ce_50_O*_x_*, Ni_25_Ce_75_O*_x_* and Ni_10_Ce_90_O*_x_* catalysts, Table S1: Atomic ratios of Ce, Ni and O in Ni*_y_*Ce_100−*y*_O*_x_* catalysts derived from XPS results, Table S2: Raman peak areas at 563 cm^−1^ of Ni*_y_*Ce_100−*y*_O*_x_* catalysts.
