# Supplementary material for: Modulating Ni/Ce Ratio in NiyCe100−yOx Electrocatalysts for Enhanced Water Oxidation
Source: Nanomaterials (Basel). 2021 Feb 9;11(2):437. doi: 10.3390/nano11020437 (PMC7914620; doi:10.3390/nano11020437)
Supplement: Supplementary file 1 [file nanomaterials-11-00437-s001.pdf]

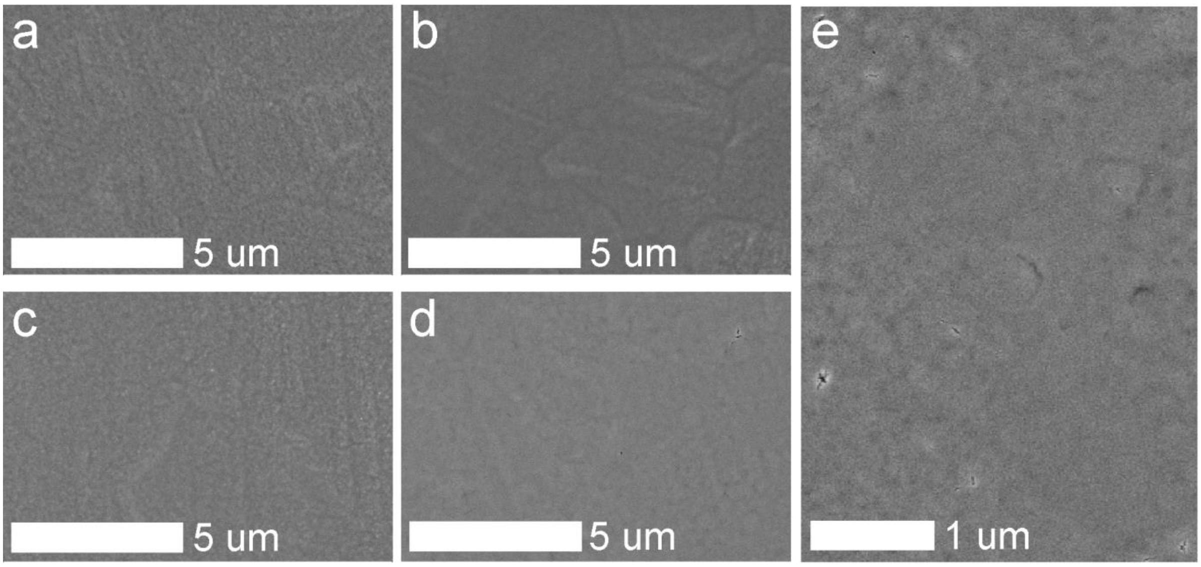

Figure S1 SEM images of (a) $Ni_{90}Ce_{10}O_x$, (b) $Ni_{75}Ce_{25}O_x$, (c) $Ni_{50}Ce_{50}O_x$, (d) $Ni_{25}Ce_{75}O_x$, and (e) $Ni_{10}Ce_{90}O_x$ catalysts.

Table S1 The atomic ratio of Ce, Ni and O in $Ni_yCe_{100-y}O_x$ samples derived from XPS results.

Catalyst	Ce %	Ni %	O %	Ni/Ce ratio
$Ni_{95}Ce_5O_x$	2.07	40.01	57.92	95/5
$Ni_{90}Ce_{10}O_x$	2.25	37.34	60.41	94/6
$Ni_{75}Ce_{25}O_x$	3.45	31.88	64.66	90/10
$Ni_{50}Ce_{50}O_x$	5.20	26.72	68.07	84/16
$Ni_{25}Ce_{75}O_x$	8.35	18.49	73.16	69/31
$Ni_{10}Ce_{90}O_x$	7.59	16.67	75.74	69/31

Table S2 Raman peak areas at 563 cm^{-1} of $Ni_{y}Ce_{100-y}O_{x}$ catalysts in **Figure 3** of the manuscript.

Catalyst	Area (*a.u.*)
$Ni_{95}Ce_{5}O_{x}$	151536
$Ni_{90}Ce_{10}O_{x}$	177345
$Ni_{75}Ce_{25}O_{x}$	237715
$Ni_{50}Ce_{50}O_{x}$	235253
$Ni_{25}Ce_{75}O_{x}$	112558
$Ni_{10}Ce_{90}O_{x}$	117829
